# Imagery May Arise from Associations Formed through Sensory Experience: A Network of Spiking Neurons Controlling a Robot Learns Visual Sequences in Order to Perform a Mental Rotation Task

**DOI:** 10.1371/journal.pone.0162155

**Published:** 2016-09-21

**Authors:** Jeffrey L. McKinstry, Jason G. Fleischer, Yanqing Chen, W. Einar Gall, Gerald M. Edelman

**Affiliations:** The Neurosciences Institute, La Jolla, California, United States of America; Rutgers The State University of New Jersey, UNITED STATES

## Abstract

Mental imagery occurs “when a representation of the type created during the initial phases of perception is present but the stimulus is not actually being perceived.” How does the capability to perform mental imagery arise? Extending the idea that imagery arises from learned associations, we propose that mental rotation, a specific form of imagery, could arise through the mechanism of sequence learning–that is, by learning to regenerate the sequence of mental images perceived while passively observing a rotating object. To demonstrate the feasibility of this proposal, we constructed a simulated nervous system and embedded it within a behaving humanoid robot. By observing a rotating object, the system learns the sequence of neural activity patterns generated by the visual system in response to the object. After learning, it can internally regenerate a similar sequence of neural activations upon briefly viewing the static object. This system learns to perform a mental rotation task in which the subject must determine whether two objects are identical despite differences in orientation. As with human subjects, the time taken to respond is proportional to the angular difference between the two stimuli. Moreover, as reported in humans, the system fills in intermediate angles during the task, and this putative mental rotation activates the same pathways that are activated when the system views physical rotation. This work supports the proposal that mental rotation arises through sequence learning and the idea that mental imagery aids perception through learned associations, and suggests testable predictions for biological experiments.

## Introduction

Mental imagery occurs “when a representation of the type created during the initial phases of perception is present but the stimulus is not actually being perceived” [1]. Such imagery may be useful to the extent that it allows internal simulation of the external world. Indeed, internal mental imagery processes can faithfully reflect corresponding processes in the world, as if the imagery were a simulation [2]. For example, humans seem to be able to rotate images of objects as if they were seeing an object rotate. In the classic experiments of Shepard and Metzler [3], human subjects were shown a pair of drawings, each depicting a single object, where the drawings sometimes depicted rotated versions of the same object. The subjects reported whether the two drawings were of the same object. There was a linear relationship between the time it took to report and the angular rotation depicted, suggesting that subjects mentally simulated the rotation of one object to the position of the other in order to make their decision. Consistent with this notion, humans seem to have mental images of intermediate rotation angles during such mental rotation [4–6]. There is extensive overlap between the brain regions active during imagery and perception of rotating objects [7], consistent with Kosslyn et al.’s [1] definition of mental imagery.

How does the capability to perform such mental rotation arise? It is known that infants as young as six months show signs of mental rotation ability [8], but how such ability develops remains unclear. It has been suggested that “implicit visual images are elicited by learned associative cues and serve to augment sensory data with ‘likely’ interpretations, in order to overcome the ever-present noise, ambiguity, and incompleteness of the retinal image” [9]. Might this idea explain how mental rotation arises? Initial sensory data of an image may recall the associated imagery of the stimulus in a slightly rotated orientation, which may in turn recall the next nearby orientation, and so forth. We suggest that mental rotation may depend on this general mechanism of associative sequence learning. During mental rotation, internally generated imagery of a rotating object could arise from regenerating the sequence of neural activity that was learned by passively observing rotating objects. Consistent with Albright’s hypothesis [9], viewing a static stimulus would allow for recalling other views of the stimulus, allowing the system to go beyond the information given [10] to solve a mental rotation task. This is consistent with the fact that parietal region activation is associated with both mental rotation and implicit sequence learning [11].

**Fig 1:**
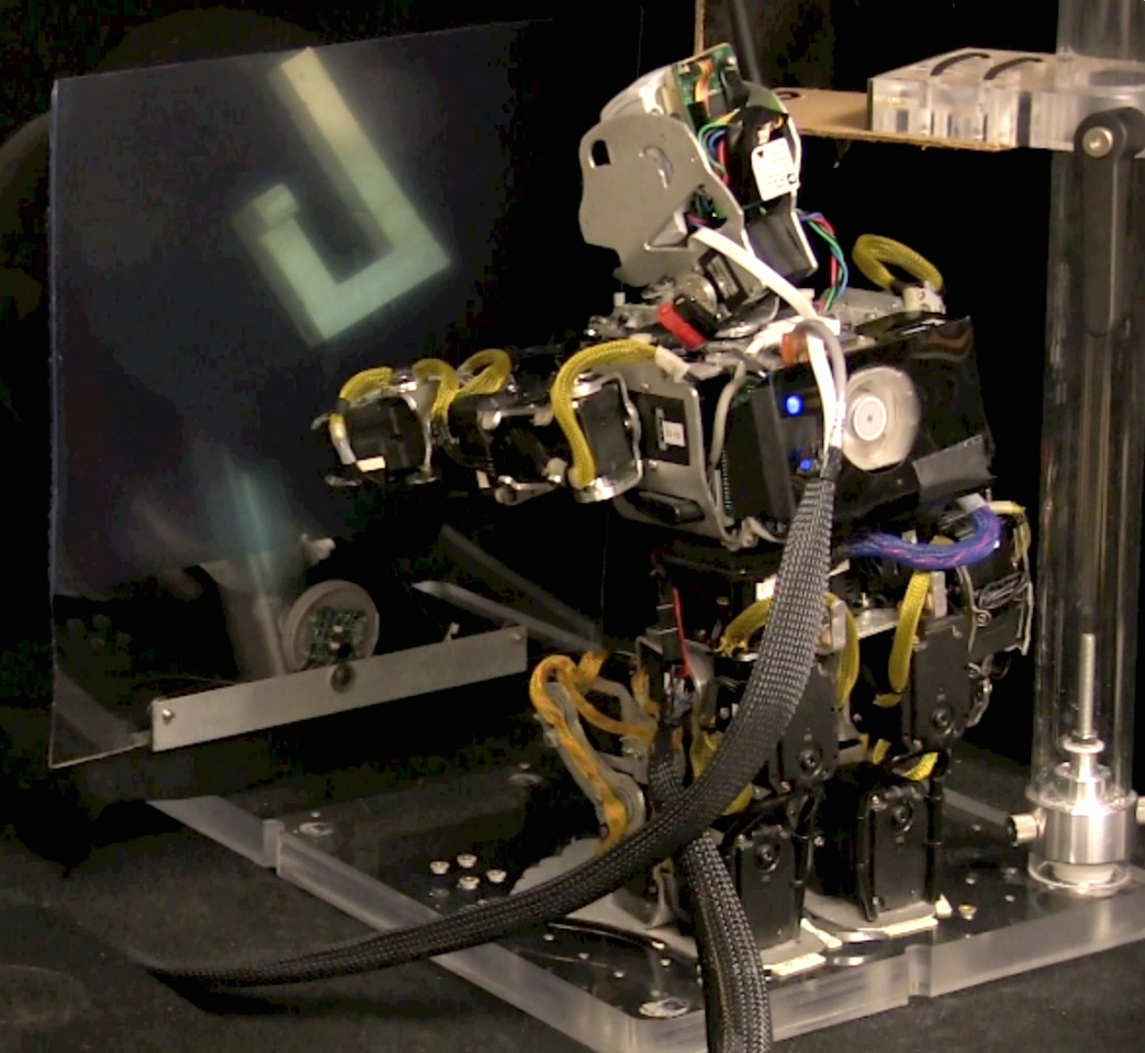
Experimental setup. Darwin XIII is positioned in front of the DMS apparatus. The apparatus can rotate the stimulus object (light-colored J-shape) to one of four selected orientations or flip sides to expose a different object that is the mirror image of the first. During the delay period and whenever the stimulus is changed, a mechanical blinder is lowered in front of the camera in the robot’s head. To indicate a “match” response, Darwin XIII reaches toward the object with its left arm to touch the clear Plexiglas panel, which activates a sensor. The time of activation is recorded for behavioral response analysis.

To test this proposal, we used the brain-based device approach [12]. A robotic device, controlled by a biologically reasonable neural model, engaged both an inner world through imagery and the real world through behavior. Extending a previous model of sequence learning, we created a large-scale, biologically detailed spiking neural model with reentrant connectivity between simulated brain regions [13,14] and used it to control a humanoid robot. The entire neurorobotic system we refer to as Darwin XIII (Fig 1). The neural model includes simulated areas for vision, sequence learning, working memory, decision making, and motor control.

Darwin XIII was challenged with a delayed match-to-sample (DMS) version of Shepard and Metzler’s mental rotation task [3] in which the subject must determine whether two objects are identical regardless of their orientations. This task provides a concrete behavioral test for mental imagery with sufficient behavioral and neuroimaging data for comparison. We demonstrate that Darwin XIII can learn to perform this mental rotation task. First, Darwin XIII was trained to report whether two stimuli were identical. Subsequently, after experience with rotating objects, Darwin XIII spontaneously generalized to report a match if two static stimuli were the same object at different orientations. Like the subjects of Shepard and Metzler, the time taken by Darwin XIII to respond was proportional to the angle of rotation between the two stimuli. In addition, the system’s neural activity during the response period was in the same activated areas, and showed the same sequence of activity patterns associated with intermediate rotation angles, as when the system was exposed to physical rotation. We do not claim that Darwin XIII is performing mental rotation as a human would, since it is a model lacking many important aspects of human physiology and behavior. However, these results support the proposal that mental rotation could arise through learned associations, support the idea that implicit imagery recalled through learned associations aids in perception [9], suggest several testable predictions, and provide a basis for future modeling studies of mental imagery.

## Methods

### Darwin XIII

We designed and constructed a humanoid robot, which, together with its neuronal network control system, we call Darwin XIII. Fig 1 shows Darwin XIII performing the mental rotation task. The device is 50 cm high, and the head contains an Axis 207MW Wi-Fi camera for vision. Each arm contains eight Dynamixel motors (Robotis, Irvine, CA, USA). In the experiments described here, only the two shoulder motors and the elbow motor of the left arm were used; all other joints remained stationary. A miniature PC (VIA Technologies, Fremont, USA) mounted on the back of the robot maintained communication between the robot and the neural simulations that ran on a Mac Pro (Apple, Inc. Cupertino, CA). We developed the simulation software in C++ using MPI libraries for parallelization and communication.

### Mental rotation task

**Fig 2:**
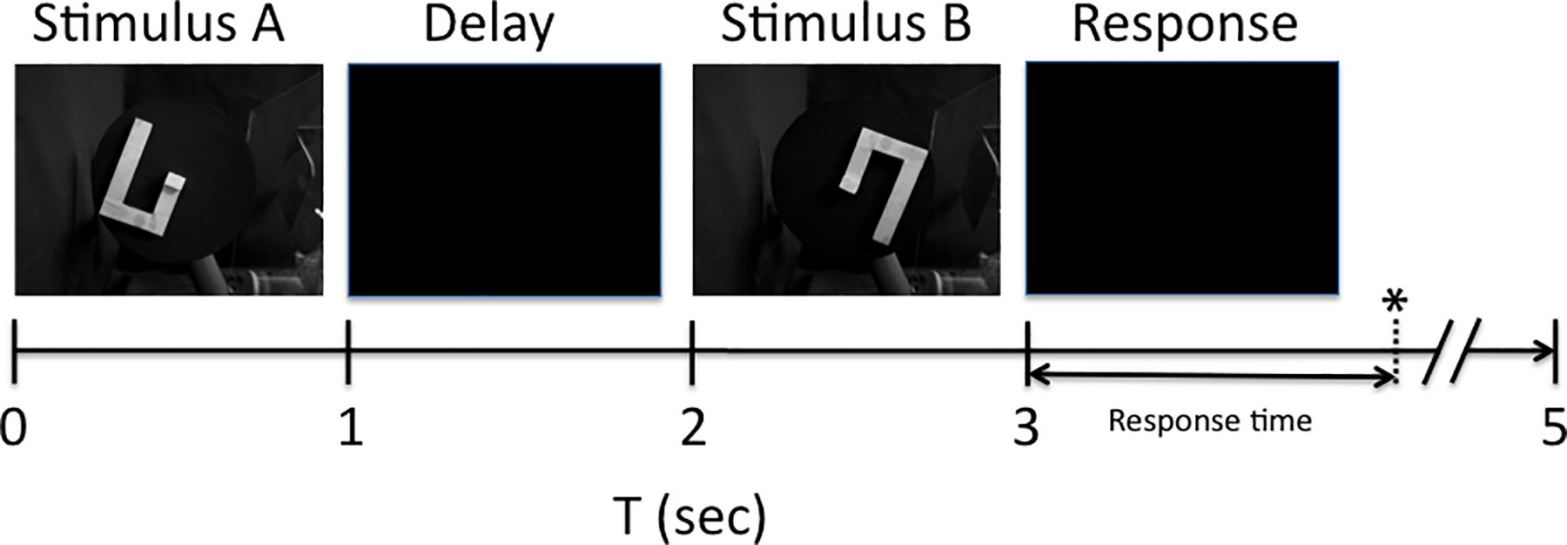
Delayed match-to-sample version of the mental rotation task. The timeline for a single trial shows the four task phases. Images of the visual input at each phase are shown above the timeline. First, Stimulus A is presented for one second. Then a blinder is lowered for a one-second delay during which the object may be switched or rotated. Stimulus B is then presented for one second, and the blinder is again lowered. Darwin XIII is given up to two seconds to respond with a movement if the objects match, as they do in this trial. The elapsed time from the offset of Stimulus B until Darwin XIII touches the Plexiglas panel (see Fig 1) is recorded as the response time.

We developed a delayed match-to-sample (DMS) version of the original Shepard and Metzler mental rotation task [3] illustrated in Fig 2. Darwin XIII is shown an object in a certain orientation for one second (Stimulus A) followed, after a one-second delay, either by the same object, which may be rotated into a different position, or by a different object, which itself may be in one of several rotated positions (Stimulus B). The second object is shown for one second, and Darwin XIII is then given up to two seconds to respond with a specific movement if the second object is the same as the first. Fig 2 illustrates a single trial in which the object is presented, rotated by 180 degrees during the delay period (when the visual input is disabled), and presented again.

**Fig 3:**
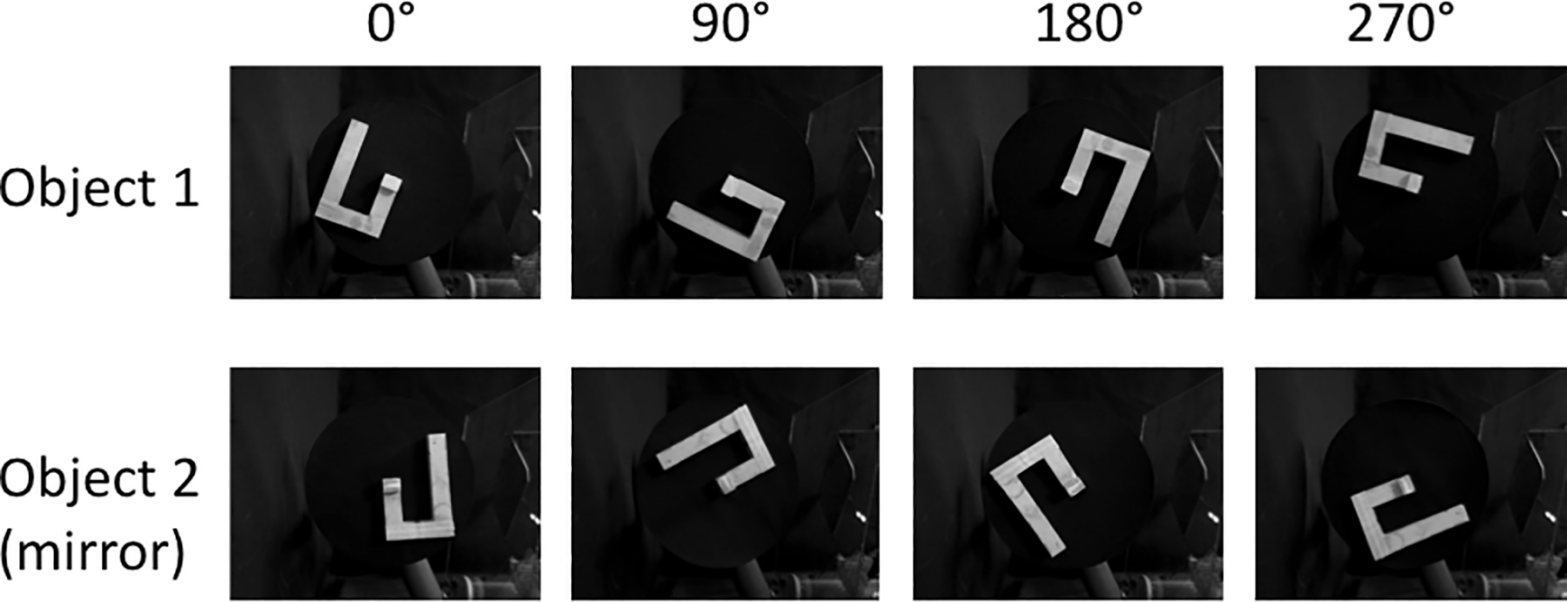
Images of the objects and rotational orientations used. The two three-dimensional wooden block constructions are related by mirror symmetry. Successive rotations are by 90 degrees in the counter-clockwise direction.

As objects, we used two three-dimensional wooden constructions that were mirror images and therefore could not be rotated to match. Each object had four possible rotations in a plane: 0, 90, 180, and 270 degrees, for a total of eight unique stimuli (Fig 3). With eight possibilities for the first object presentation (Stimulus A) and a similar eight possibilities for the second object presentation (Stimulus B), there were 64 unique trials possible. Half show the same object for both stimuli, called “match” trials, and half show different objects, or”non-match” trials.

### Network and neuron model

**Fig 4:**
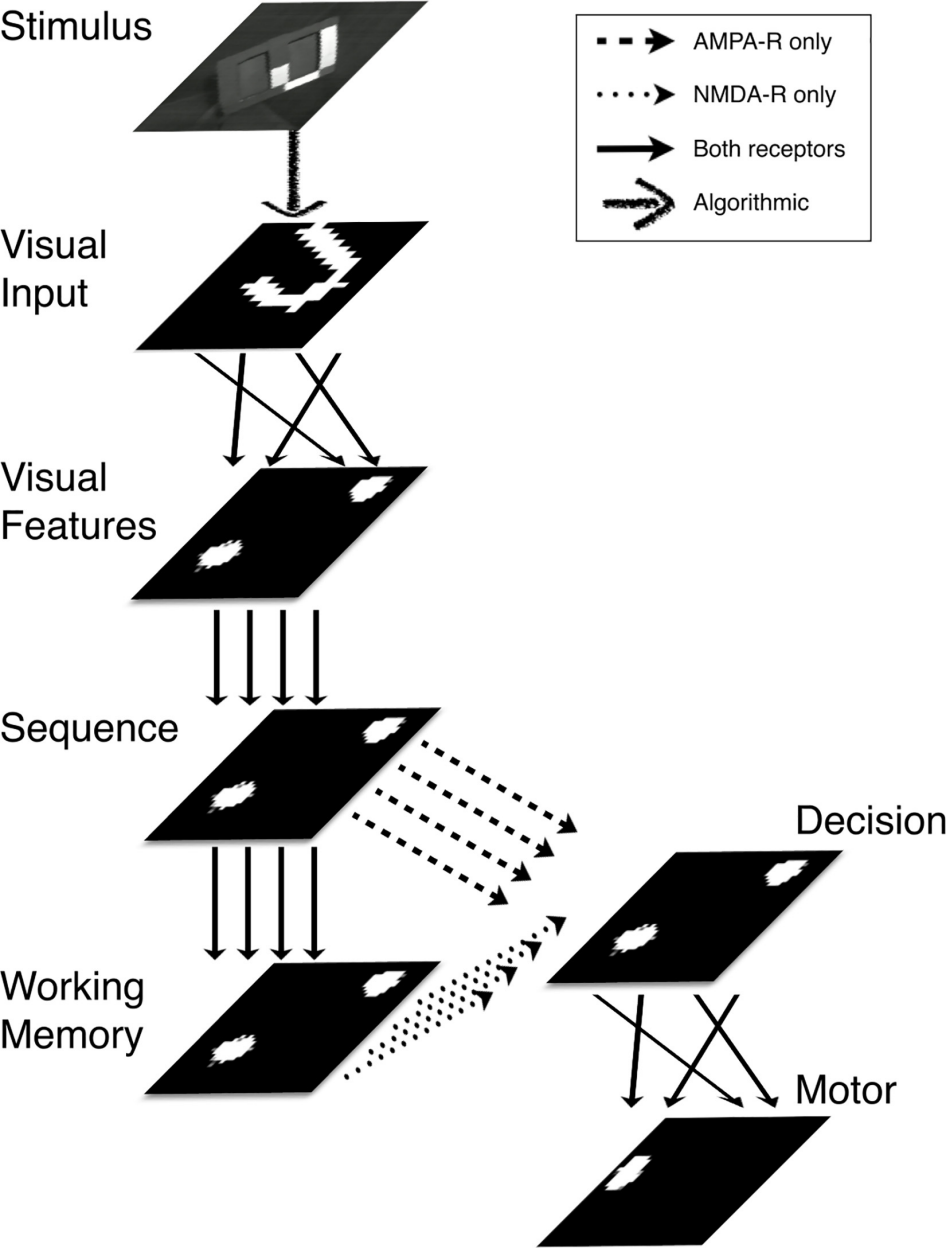
Simulated nervous system that learns to perform the mental rotation task through sequence learning. The neural areas, represented by labeled rectangles, consist of two-dimensional winner-take-all networks of excitatory and inhibitory spiking Izhikevich neurons [14,15]. The arrows show the excitatory neural pathways between the areas. Parallel arrows indicate topographic connectivity, while crossing arrows indicate non-topographic connectivity. The figure illustrates the moment of matching when it performs mental rotation in the DMS task. Bright spots indicate active groups of excitatory neurons within each of the neural areas. Presentation of Stimulus A causes neural responses in the Visual Input area, the Visual Features area, the Sequence area, and the Working Memory area. During the delay period, the Working Memory area maintains its activity pattern while the Visual Input and Visual Features areas become silent. Presentation of Stimulus B causes activity in the Visual Input, Visual Features, and Sequence areas, while the Working Memory area maintains the Stimulus A pattern. When Stimulus B is removed, the Sequence area cycles through activity patterns that it learned by experiencing rotating objects. Because Decision area afferents contact separate receptor types (AMPA or voltage-gated NMDA), it responds if and only if the activity pattern in the Sequence area comes to match that in Working Memory, as shown in the figure. After training, it responds to matching stimuli, Decision area firing activates Motor area neurons, causing the robot to reach out and touch a Plexiglas panel.

To perform the mental rotation task, the simulated neural network has six basic functions that are implemented in different neural areas: (1) retinotopic visual processing, (2) encoding features of visual inputs in non-retinotopic neural activity patterns, (3) learning sequences of visual feature activity patterns and regenerating those sequences when cued, (4) maintaining a visual feature activity pattern after the stimulus encoded is gone, (5) deciding if a match exists between the current visual feature activity pattern and the one held in memory, and (6) triggering a behavioral response to signal the match. Fig 4 shows a block diagram of the system.

Except for the Visual Input area, each area is a winner-take-all network of excitatory and inhibitory neurons [13,14]. Five neuron types were simulated using the Izhikevich spiking neuron equations [15] and the parameters listed in Table 1. For each neuron type in an area, Table 2 lists the number of neurons, the average total number of afferent synapses per neuron, and the source area, neuron type, and percentage distribution for the different presynaptic connections arriving on such a neuron. Within areas, neurons were connected on a two-dimensional sheet with a Center-Annular Surround anatomy [13] that effectively generates winner-take-all dynamics. Between areas, neurons were connected topographically, with probability distributions in the target area determined by a circular Gaussian centered on the location corresponding to that of the projecting neuron. The minimum and maximum radii and standard deviation of the circular Gaussian is given for each projection in Table 2. In order to avoid boundary conditions, each area was treated as a torus, with connections from neurons on one edge “wrapping around” to connect with neurons on the other edge.

**Table 1:** Parameters for the five types of neurons in the simulations and the neural areas in which they are used. The nine parameters, applied in the Izhikevich spiking neuron equations [16], determine the spiking behavior of the simulated neurons (see [14]).

Neuron type (abbreviation)	Area	C (pF)	k (a.u.)	v_r_ (mV)	v_t_ (mV)	v_peak_ (mV)	a (a.u.)	b (a.u.)	c (mV)	d (pA)
Thalamic Input (Thal)	Visual Input	200	1.6	-60	-50	40	0.01	15	-60	10
Excitatory, Layer 2/3 (E2/3)	Visual Features, Sequence,Working memory, Decision	80	3	-60	-50	50	0.01	5	-60	10
Excitatory, Layer 5/6 (E5/6)	Visual Features, Sequence,Working memory	80	3	-60	-50	50	0.01	5	-60	10
Inhibitory, Layers 2/3 and 5/6 (I2/3, I5/6)	Visual Features, Sequence,Working memory, Motor	20	1	-55	-40	25	0.15	8	-55	200
Excitatory, Layer 5/6 (E5/6M)	Motor	100	0.7	-60	-50	0	0.03	-2	-60	100

**Table 2:** Parameters for composition of neural areas, connectivity, and learning in Darwin XIII. The neural areas area assumed to be 2mm by 2mm toroidal sheets for the purposes of the connectivity distributions described here. See [14] for detailed descriptions of these parameters.

Neural area	Neuron type	Number of neurons	Synapse parameters				Topological connectivity parameters			Synaptic scaling parameters		Synaptic currents			STDP Learning rate		Learning phase	
			Average synapses per neuron	Pre-synaptic neuron area	Pre-synaptic neuron type	% of total synap-ses	r_min_ mm	r_max_ mm	σ mm	s_total_ nS	s_max_ nS	*gain*_*AMPA*_	*gain*_*NMDA*_	*gain*_*NMDAVI*_	Initial α	Final α	Learning start time (ms)	Learning end time (ms)
Stimulus	Thal	441																
Visual	E2/3	1849	4400	Visual	E2/3	10	0	0.1	0.05	22	10	1	0.5	0				
				Visual	I2/3	20	0.1	1	0.8	1200	20	1	0.5	0				
				Stimulus	Thal	50	0	1.44	10	200	50	1	0.5	0	0.002	0.3	0	100000
				Sequence	E5/6	20	0.35	1	0.35	20	6	1	0	0.25	0.9	0.9	810000	904000
	I2/3	900	1600	Visual	E2/3	25	0	0.33	0.16	100	20	1	0.5	0				
				Visual	I2/3	50	0.1	1	0.8	180	30	1	0.5	0				
				Stimulus	Thal	25	0	4	10	10	10	1	0.5	0				
	E5/6	1849	1980	Visual	E5/6	12	0	0.1	0.05	30	10	1	0.5	0				
				Visual	E2/3	44	0	0.1	0.05	22	20	1	0	0.5				
				Visual	I5/6	44	0.1	1	0.4	900	30	1	0.5	0				
	I5/6	441	1200	Visual	E5/6	33	0	0.33	0.16	100	2	1	0.5	0				
				Visual	I5/6	67	0.1	1	0.4	180	3	1	0.5	0				
Sequence	E2/3	1849	4400	Sequence	E2/3	10	0	0.1	0.05	22	10	1	0.5	0				
				Sequence	I2/3	20	0.1	1	0.8	1200	20	1	0.5	0				
				Visual	E5/6	50	0	0.1	0.05	200	50	1	0.5	0	0.002	0.4	100000	140000
				Sequence	E5/6	20	0.35	1	0.35	150	30	1	0	0.5	0.9	0.9	810000	904000
	I2/3	900	1200	Sequence	E2/3	33	0	0.33	0.16	100	20	1	0.5	0				
				Sequence	I2/3	67	0.1	1	0.8	180	30	1	0.5	0				
	E5/6	1849	1980	Sequence	E5/6	10	0	0.1	0.05	40	20	1	0.5	0				
				Sequence	E2/3	45	0	0.1	0.05	22	20	1	0	0.5				
				Sequence	I5/6	45	0.1	1	0.4	900	30	1	0.5	0				
	I5/6	441	1200	Sequence	E5/6	33	0	0.33	0.16	100	2	1	0.5	0				
				Sequence	I5/6	67	0.1	1	0.4	180	3	1	0.5	0				
Working memory	E2/3	3136	1430	WM	E2/3	15	0	0.1	0.03	50	45	1	0.5	0				
(WM)				WM	E2/3	77	0.1	1	0.4	900	60	1	0.5	0				
				Sequence	E5/6	8	0	0.1	0.03	7	7	1	0.5	0				
	I2/3	784	1200	WM	E2/3	33	0	0.33	0.16	80	2	1	0.5	0				
				WM	I2/3	67	0.1	1	0.4	180	3	1	0.5	0				
Decision	E2/3	3136	3740	Decision	E2/3	10	0	0.1	0.05	22	10	1	0.5	0				
				Decision	I2/3	30	0.1	1	0.333	900	10	1	0.5	0				
				Sequence	E5/6	30	0	0.1	0.05	0.725	1	1	0	0				
				WM	E2/3	30	0	0.1	0.05	0.725	1	0	1	0				
	I2/3	784	2200	Decision	E2/3	18	0	0.33	0.16	15	2	1	0.5	0				
				Decision	I2/3	46	0.1	1	0.333	180	3	1	0.5	0				
				Sequence	E2/3	18	0	1.44	10	10	2	1	0.5	0				
				WM	E2/3	18	0	1.44	10	10	2	1	0.5	0				
Motor	E5/6	1600	3200	Motor	E5/6	12	0	0.33	0.16	5	5	1	0.5	0				
				Motor	I5/6	24	0.333	1.44	2.5	1200	100	1	0.5	0				
				Decision	E2/3	64	0	1.41	4.5	150	50	1	0.5	0				
	I5/6	400	1000	Motor	E5/6	20	0	0.33	0.16	10	5	1	0.5	0				
				Motor	I5/6	40	0.333	1.44	2.5	800	20	1	0.5	0				
				Decision	E2/3	40	0	1.41	4.5	40	10	1	0.5	0				

The number of synapses for each projection was scaled from the same probability density function, and the initial synaptic strengths were set using the same function and parameters. However, the sum of all synaptic strengths of each type was scaled to make the total equal to a constant value (S_total_), with a maximum strength (S_max_) for individual synapses (Table 2). The parameters for the AMPA, NMDA, and NMDA voltage independent conductances are also given in Table 2. Other conductances and short-term synaptic plasticity (STSP) parameters were the same as described in McKinstry and Edelman [14], except for the decision area, where STSP was disabled for all targets of the excitatory neurons.

Some connection strengths were adjusted using a spike-timing-dependent plasticity (STDP) rule, whereby presynaptic spikes occurring within a brief time window prior to postsynaptic spikes trigger long-term potentiation, and the reverse spike order triggers long-term depression as previously described [14]. As given in Table 2, a learning rate governed the application of STDP on certain pathways during network training stages specified by the learning phase start time and end time columns. For convenience, we employ three phases of learning, as indicated in the last 2 columns of Table 2. Such a strategy is reasonable in a hierarchical visual system, where later stages depend upon the appropriate output of earlier areas. Such staging may occur in the brain analogous to so-called critical periods. In the first phase the visual area learns to discriminate patterns. In the second phase, connections from the visual area to the sequence area are strengthened. Finally, in the last phase, the sequence area learns specific sequences based upon stable pattern responses in the visual area.

### Operation of the system

The central portion of Darwin XIII’s camera image was used to drive the 21x21 array of neurons in the Visual Input area. The receptive fields of adjacent neurons were mapped onto adjacent image pixels, based on a model of on-center/off-surround retinal ganglion cells with a 3x3 pixel on-center area and a 6x6 pixel off-surround area. At each numerical integration step in the simulation, each neuron received a current injection that was proportional to the intensity of the pixels in its receptive field; see [14].

After training (see below), the Visual Features area displayed unique activity patterns in response to each of the eight visual stimuli. When a stimulus is shown, the unique patterns in the Visual Features area activate the corresponding locations in the Sequence area through the topographic connections between the two areas. After undergoing training to learn a sequence of visual stimuli (see below), Sequence area activity would repeatedly cycle through the entire learned sequence in response to a brief presentation of any one of the stimuli [14]. The outputs of layer 5/6 neurons in the Sequence area provided topographic input to the Working Memory area and to the Decision area.

During each DMS trial, the Working Memory area maintained the pattern corresponding to that of the Sequence area during the presentation of Stimulus A and maintained that pattern through the remainder of the five-second trial. Local connections within the Working Memory area are tuned to support the formation of persistent activity through attractor-like dynamics. These persistent firing patterns are generated by strong local excitation and strong annular inhibition (Table 2). Thus, Working Memory area acquires a copy of the Stimulus A pattern from Sequence area inputs, and it then maintains the Stimulus A firing pattern after the activity pattern in the Sequence area has changed in response to Stimulus B.

During the presentation of Stimulus B, the Sequence area responded with the pattern appropriate to that stimulus. When Stimulus B was removed, the Sequence area proceeded to cycle through the learned activity patterns for that object, beginning with the one corresponding to Stimulus B. Working Memory area does not pick up a copy of the Stimulus B pattern due to lateral inhibition from the Stimulus A pattern.

Neurons within the Decision area responded only when they received topographic input simultaneously from the corresponding regions of the Sequence and Working Memory areas. Afferents from the Working Memory area connect to synapses with NMDA receptors, while afferents from the Sequence area connect to synapses with AMPA receptors (detailed parameters are given in Table 2). Because the NMDA receptor is voltage gated, persistent activity in the Working Memory area would generate large NMDA receptor activation, but could not by itself cause Decision area neurons to spike. Sequence area inputs were tuned to be too weak to cause firing by themselves. However, if a Decision area neuron with activated NMDA receptors (input from Working Memory area) is depolarized by the action of AMPA receptors (input from Sequence area), it will begin to fire. Thus any spiking activity in the Decision area indicates a match between the stimulus “remembered” through persistent activity in the Working Memory area and the currently presented stimulus that causes the activity pattern in the Sequence area.

Neurons in the Decision area connect widely to those in the Motor area, which controls the left arm of Darwin XIII. After the training of the motor response, as described below, activity in the Decision area caused Darwin XIII to extend its left arm, indicating a match. At the end of each trial, the voltages and conductances of neurons in the Working Memory and Decision areas were reset to a resting state so that they could respond again on the subsequent trial.

### Training procedures

In the first stage of training, the Visual Features area developed unique activity patterns for each of the eight stimuli (Fig 3); the patterns were unique due to the winner-take-all competition within the network and STDP applied to connections coming from the Visual Input area [13]. The first stimulus was presented for one second, followed by the second stimulus for one second, and so on in a repeating pattern for a total of 100 simulated seconds. After this training period, STDP was disabled on those weights. Then, STDP was enabled in the pathway from the Visual Features area to the Sequence area, and each stimulus was presented five additional times for one second each for a total of 40 seconds (Table 2), thus fine-tuning the synaptic strengths in that pathway.

In the second stage of training, Darwin XIII learned to provide a behavioral response to indicate whether the stimuli matched during a DMS trial. Connections between the Decision area and the Motor area were strengthened by simulated neuromodulation in a reinforcement learning paradigm. Eight identical stimulus pairs (same object, same orientation) and eight non-identical pairs (different objects, same orientation) were presented four times each during training for a total of 64 trials. Initially, the connection strengths were set at a low level so that activity in the Decision area did not activate neurons in the Motor area. On half the trials at random, current was injected into a small patch of neurons within the Motor area, causing the left arm to move toward the object and touch a Plexiglas panel. On a trial when the stimuli were identical and Darwin XIII happened to respond correctly by chance, simulated dopaminergic neurons were activated prompting dopamine-dependent STDP [16] potentiating those synapses that could elicit the conditioned response when the Decision area signaled a match. Following the training, random current injection in the Motor area was disabled, and any behavioral responses were generated only through the activity within the Decision area.

In the third stage of training, the Sequence area learned to generate a succession of activity patterns that reflected a sequence of sensory experiences. Each object was presented to Darwin XIII in four different orientations reflecting the counter-clockwise rotation of the object in four steps through 360 degrees (Fig 3). Each sequence was presented five times in a row, with STDP enabled for excitatory connections within the Sequence area and for the reentrant connections to the Visual Features area. During the transition between stimuli activity patterns reflecting both stimuli were briefly co-active, enabling associative learning. When this training was complete, the brief presentation of an object in any orientation caused the activity pattern in the Sequence area to proceed continuously through the series of patterns for that object, beginning with the pattern for the orientation presented.

### Analysis of activity patterns

To compare population activity patterns under different conditions, firing rates in 50 ms windows were used to construct a population vector for each condition. The similarity in the population vectors was measured using the normalized dot product method to determine a match score ranging from zero to one, where a score of one indicates identical population vectors [14].

## Results

The entire training and testing procedure was repeated with five “subjects,” each being a different instantiation of the neural model with the same connectivity probability distributions but differing from each other in the random number seeds used to generate the detailed connectivity. The overall results were highly consistent among the subjects.

### Darwin XIII learned to distinguish identical and non-identical pairs of stimuli

A prerequisite for success in the DMS version of the mental rotation task is for Darwin XIII to be able to report whether two stimuli match. Thus Darwin XIII was trained using identical and non-identical stimuli in the DMS protocol as described above (see Methods). All five subjects were then tested on each of the 16 pairs of the training set, and Darwin XIII responded correctly to 79 of the 80 presentations.

As a control, it was important to test whether Darwin XIII would give a match response to rotated versions of the same object before it had any experience viewing rotating objects (see Methods). Therefore, with learning disabled, each subject was presented with all 64 possible stimulus pairs. The five subjects gave a match response in 39 of the 40 trials (98%) in which the two stimuli were identical, as expected. They also correctly gave a non-match response in 276 of the 280 trials (99%) where the stimuli were not identical, including the 120 trials where the second stimulus was a rotated version of the first.

### After experience with rotating objects, Darwin XIII spontaneously performed a mental rotation task

After training the Sequence area with rotating objects (see Methods), Darwin XIII was tested in the DMS task as before, using all 64 possible stimulus pairs. Overall, the five subjects responded correctly on 294 of 320 trials (92%). They performed correctly on 154 of the 160 match trials (96%), where the two stimuli were the same object, including the 120 trials where the second was a rotated version of the first.

It is important to note that Darwin XIII had never been trained to perform mental rotation, but instead was trained to signal matching stimuli and given experience viewing physical rotation of stimuli. As stated above, Darwin XIII was unable to perform mental rotation in a control experiment before viewing physical rotation. Additionally, we can rule out conditioning during the task as a possible explanation for mental rotation since all learning was disabled during the DMS task. Thus, the system was able to generalize from its initial training and, only after experience with rotating objects, spontaneously identify two views of the same object in different orientations as matching.

### Reaction times increased with angular difference between the two matching stimuli

**Fig 5:**
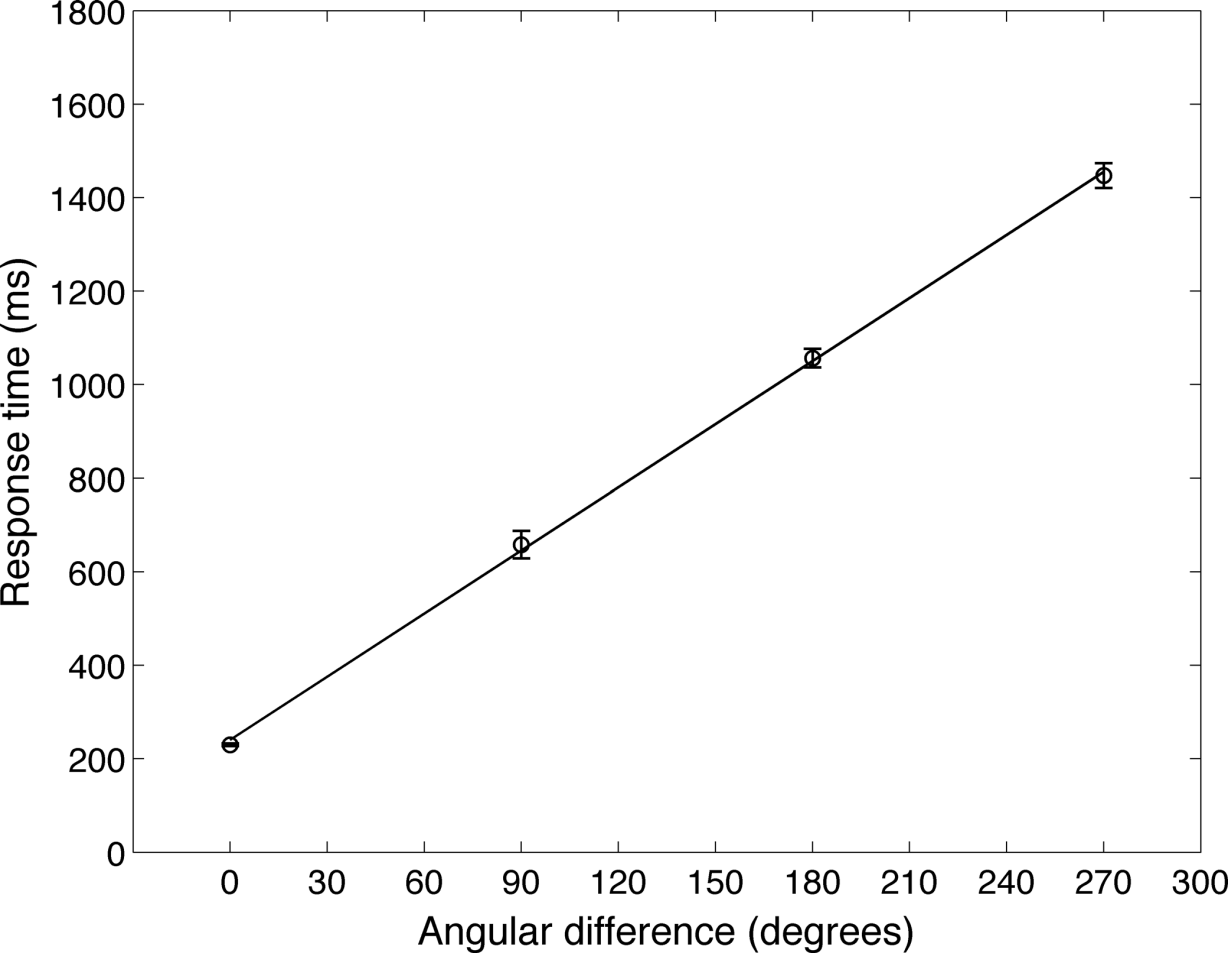
Behavioral evidence for mental rotation in Darwin XIII. Response time of Darwin XIII during match trials from a mental rotation experiment. The mean and standard deviations of response times from five different subjects is plotted as a function of the angular difference between the stimuli. The time to respond to a pair of images of the same object was proportional to the degree of angular rotation of the object between the two views, which matches the key finding from Shepard and Metzler’s study of human subjects [3].

Shepard and Metzler [3] found a linear increase in subject reaction times proportional to the angular difference between the two matching stimuli in the mental rotation task. We looked for a similar relationship in our system. Fig 5 shows the mean and standard deviations of the response times on the 160 match trials, measured behaviorally as the time from the offset of Stimulus B (T = 3 sec in Fig 2) to the time the panel was pressed (“Response” in Fig 2). The time to report a match was proportional to the angle of rotation between the two stimuli, as was the case for the human subject data.

### Internally generated activity during the DMS task progressed through patterns corresponding to intermediate rotation angles

By presenting human subjects with two successive images of an object rotated in space, an illusion of smooth motion can be induced (e.g., [17]). Consistent with this illusory perception, fMRI demonstrated cortical activity related to the unseen intermediate object orientations, an internal filling-in [6]. Such filling-in also takes place in Darwin XIII’s Sequence area during the DMS task.

**Fig 6:**
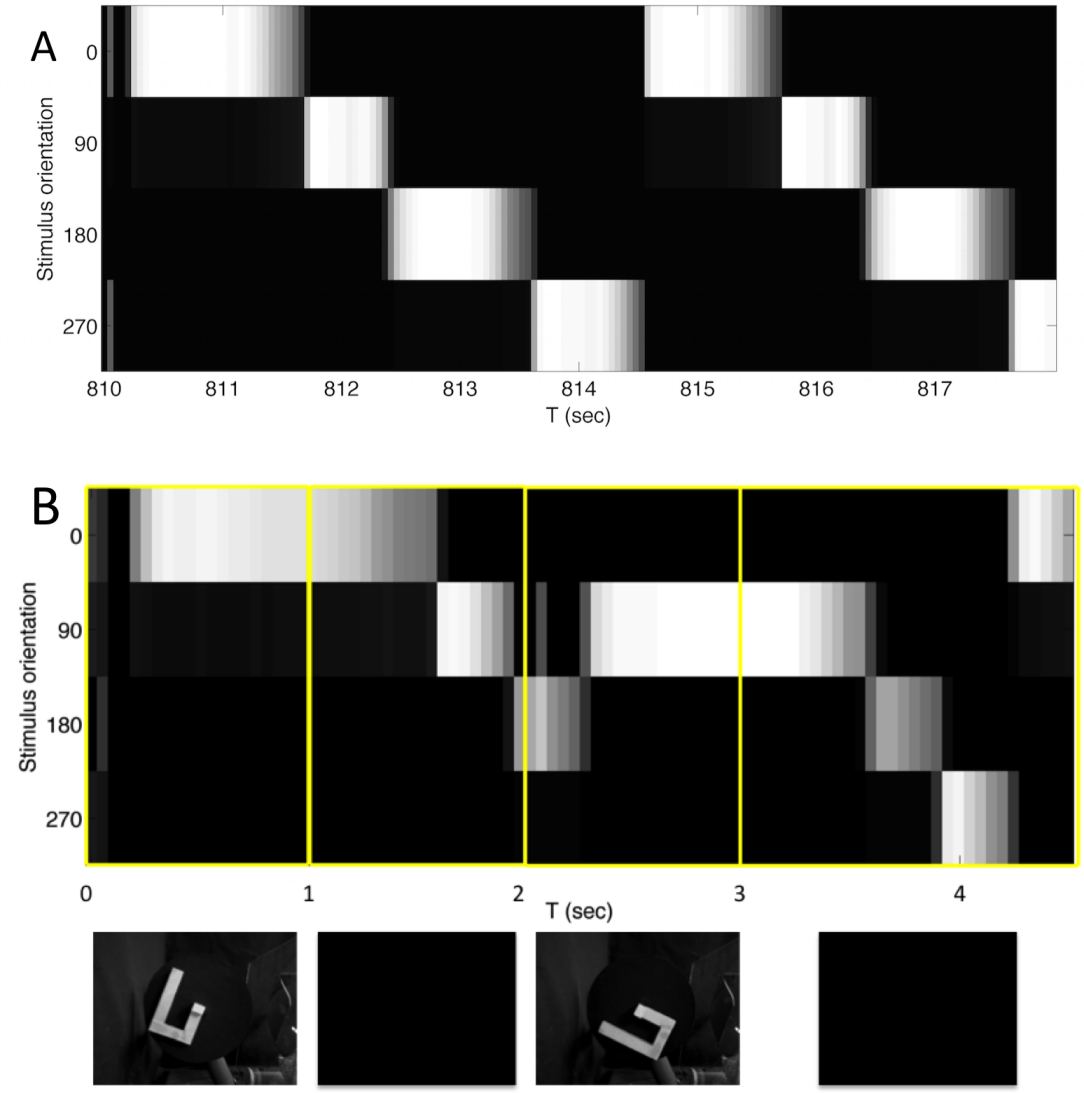
During a mental rotation task, the Sequence area fills in intermediate angles between the rotated stimuli and has activity similar to that when viewing a rotating object. (A) Sensory response when viewing a rotating object. The population activity in the Sequence area was recorded (for each 50 ms time period) as Darwin XIII was presented with Object 1 rotating through all four orientations (see Fig 3) in rotational sequence for two complete cycles. The brightness of the line at each point indicates the value of the match score (white = 1, black = 0) between the activity pattern at the time shown on the abscissa and the reference average activity pattern evoked, prior to sequence training, by static presentation of the object at the orientation shown on the ordinate. (B) Responses during the mental rotation task demonstrate filling-in and are similar to (A) in the delay and response periods. The plot, similar to that in A, shows the match scores in the Sequence area during a single trial of the DMS mental rotation task (see Fig 2; trial phases are indicated by the yellow boxes). Images of the visual input at each phase are shown beneath the plot. Stimulus A (T = 0 to 1 sec) was the object at zero degree orientation. During the Delay period (T = 1 to 2 sec), in the absence of an external stimulus, the activity pattern began to cycle. Presentation of Stimulus B (T = 2 to 3 sec), which was the same object at the 90 degree orientation, caused the activity pattern to change to the one reflecting that orientation. When the stimulus was removed (T = 3 sec) at the beginning of the Response period, the activity pattern first continued to reflect Stimulus B and then began to cycle through the sequence of patterns, filling in intermediate angles in the learned series of object orientations until the pattern matched that of Stimulus A, thus triggering Decision area activity (not shown) and a motor response. Internally generated Sequence area activity corresponded to past experience with the physical rotation of the object (as in part A of this figure).

To demonstrate this filling-in, Fig 6 illustrates the neural responses in the Sequence area both while the stimulus is present (Fig 6A), and when it is not present during the mental imagery task (Fig 6B). Fig 6A shows the match scores for activity in the Sequence area as Darwin XIII viewed the physical rotation of an object through two complete rotations. The brightness indicates the degree to which the activity pattern at that point in time corresponded to the average activity patterns evoked by static presentations of the object at each one of the four orientations. White indicates a perfect match, and black indicates no overlap. As expected, the activity patterns cycled through those corresponding to the static orientations with a high degree of fidelity.

Fig 6B shows a similar plot as Darwin XIII performed a single match trial in the mental rotation task. The four yellow boxes mark the phases of the trial as indicated in Fig 2. The visual input at each phase is shown at the bottom of the figure. After the object was presented at the 90 degree orientation as Stimulus B (T = 2 to 3 sec), the network filled-in activity (T = 3.5 to 4.5 sec) corresponding to the intermediate orientations of 180 and 270 degrees prior to triggering a match response when it reached the pattern corresponding to the 0 degree orientation, which had been presented as Stimulus A (T = 0 to 1 sec).

**Fig 7:**
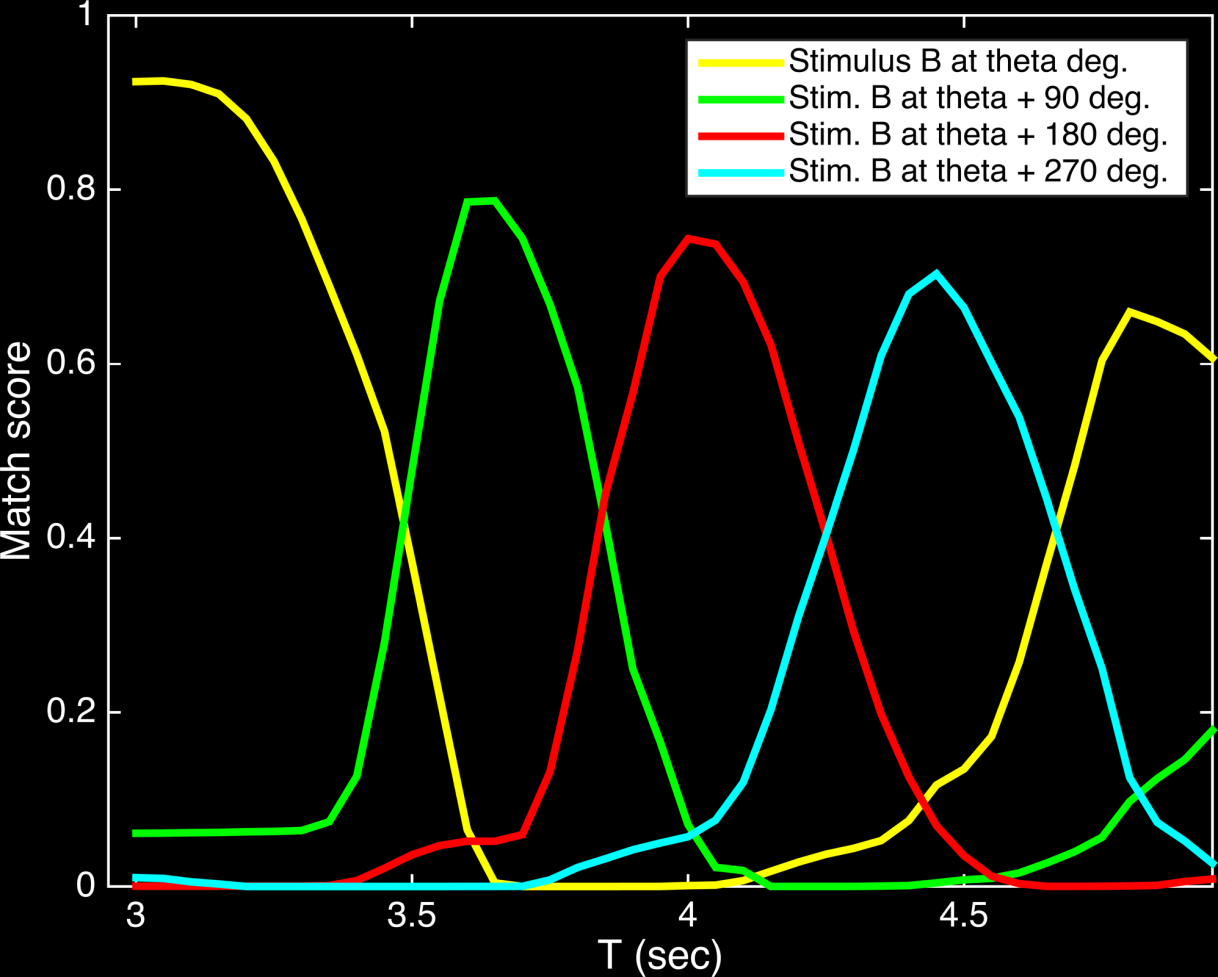
Population activity in the Sequence area during the Response period occurs in the same order as when viewing a rotating object. The match score indicates the similarity of the activity pattern at each time point to the reference activity patterns for a given object orientation. Reported match values are averaged over all subjects and all trials. Each colored line shows the match in relation to one of the four orientations, and the progression of peaks (starting at angle theta, the orientation of Stimulus B) in the colored lines indicates that the activity patterns recapitulate the sequence learned when viewing a counter-clockwise rotating object.

Fig 7 shows the average match score for the activity in the Sequence area during the response period (T = 3 to 5 sec) over all trials for all five subjects. The results clearly indicate that the activity progressed through the sequence of patterns corresponding to counterclockwise rotation through the four orientations, showing that the network fills-in intermediate orientations.

### The time course of neural responses generates the linear increase in reaction time with angular difference

**Fig 8:**
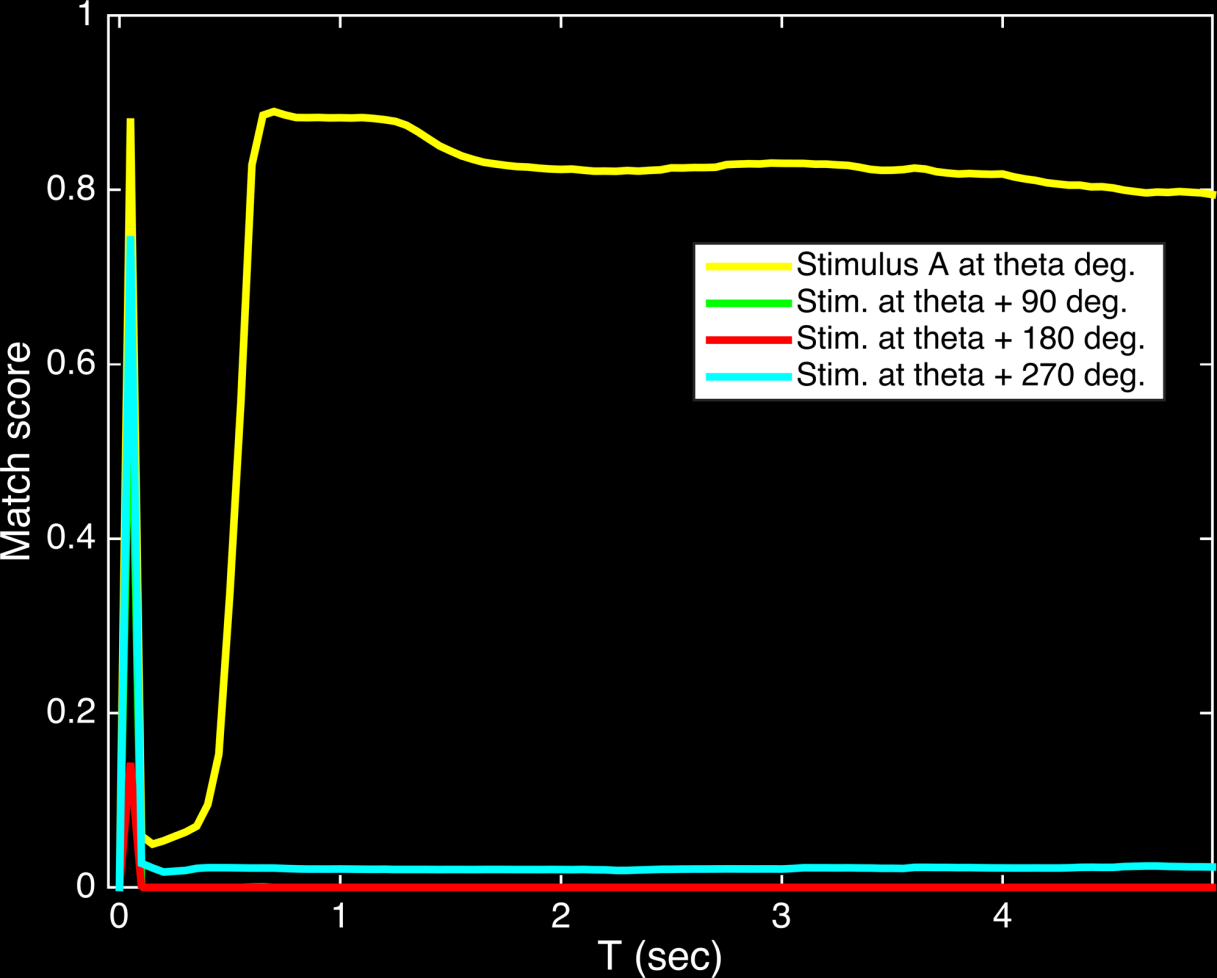
Working Memory maintains the activity pattern corresponding to Stimulus A throughout the trial. The yellow line shows the average match score, across subjects and trials, that reflects the similarity of the Working Memory activity pattern at each point in time to the reference activity pattern for stimulus A at orientation theta. Note that the low match score for the other stimulus angles indicates that working memory stores only stimulus A, the first stimulus presented in each trial (see Fig 2).

**Fig 9:**
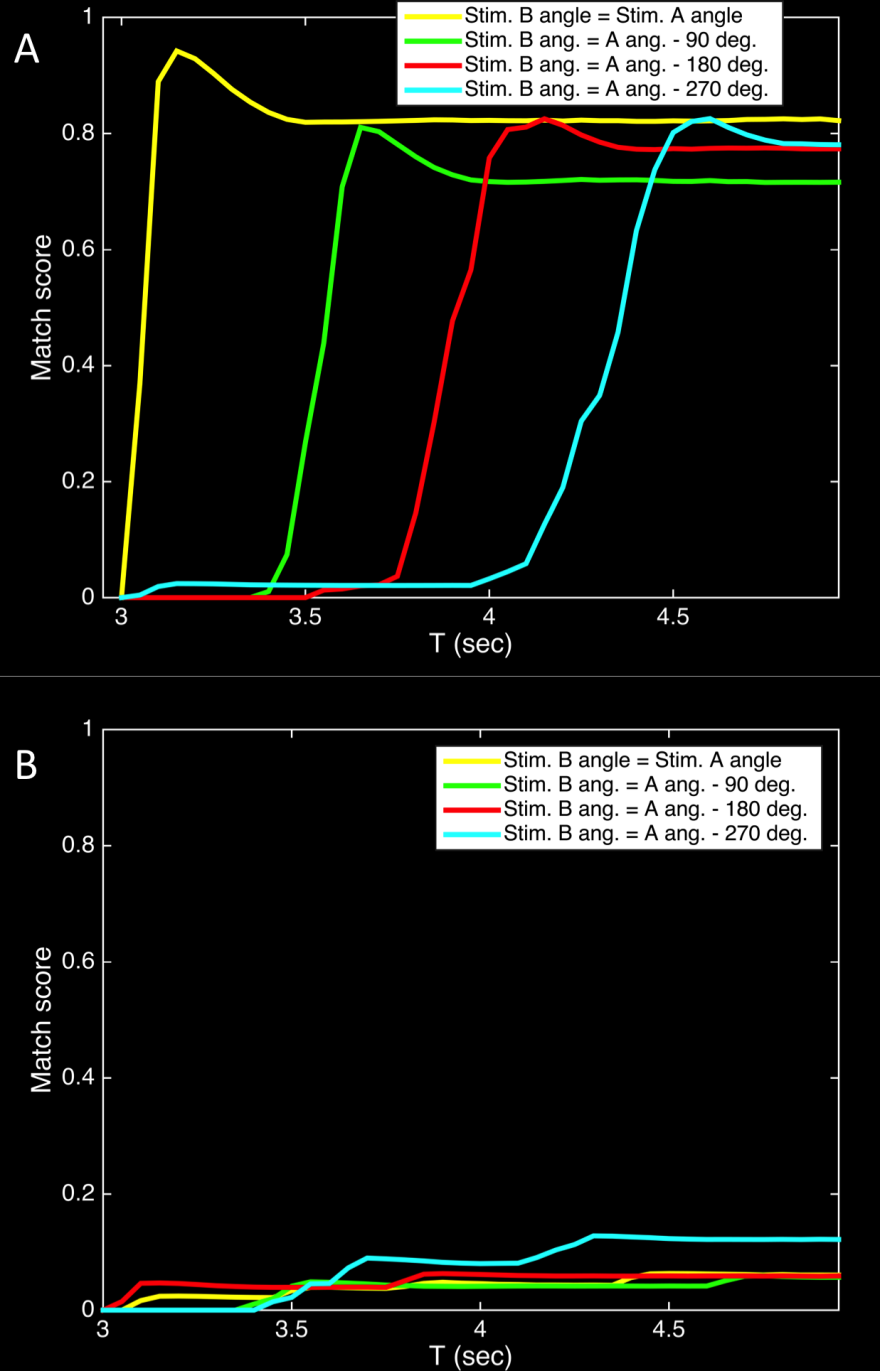
The time course of activity patterns in the Decision area during the Response period of match trials causes the linear relationship between response time and angular difference. (A) Match trials. The yellow line reflects the time course of the match score, averaged across subjects for all trials in which the stimulus B angle matched the stimulus A angle. The Decision area became active when the Sequence area pattern (see Fig 7) progressed in the cycle to the one matching that in Working Memory. The response times were progressively longer as the angle between Stimulus A and B increased (green, red, and cyan lines). (B) Non-match trials. Low match scores for all patterns in the Decision area during non-match trials indicating low overall activity, as expected, resulted in Darwin XIII correctly withholding a motor response on most non-match trials.

The Working Memory area maintained the pattern of activity corresponding to Stimulus A over the course of the trial (T = 0.5 to 5 sec; Fig 8). This produces NMDA receptor activation in corresponding Decision area neurons (see Methods). Once a corresponding pattern arises in the Sequence area it generates a small depolarization that results in spiking due to the voltage-gated NMDA conductance accumulated during the delay period. This is reflected in the activity patterns of the Decision area during the response period for match trials (Fig 9A). In the match trials, the Decision area became active shortly after the Sequence area pattern progressed (cf. Fig 7) to the one that matched the pattern in the Working Memory area. In non-match trials (Fig 9B), weak activation of the Decision area due to noisy activity patterns is a potential source of infrequent false-positive errors in behavior.

The timing of this Decision area response explains the linear relationship of reaction time with angular difference in Fig 5. When the angular difference between Stimulus B and Stimulus A was larger, the Sequence area would progress through more intermediate patterns before it could depolarize Decision area neurons to generate the behavioral response.

### Neural activity is similar in the sensory and imagery conditions

Kosslyn’s definition of mental imagery suggests that neural activity during perception and imagery should be similar. Indeed, evidence from brain imaging for such an overlap has been reported [7,9,18]. The patterns of activity in the Sequence area of the model are highly similar in the two conditions, as indicated by the match scores (e.g., Fig 6). However, while the match score is sensitive to the relative pattern of neural activity over the population, it is relatively insensitive to the absolute firing rates of individual neurons.

To address this, we compared the mean firing rates of all 20,359 neurons in the model during the DMS task, prior to the sequence training, with the corresponding rates during the mental rotation task. For this comparison, only the trials in which Stimulus A was identical to Stimulus B were used in order to keep stimuli and motor responses identical across the two conditions. Neurons with fewer than one spike per second on average were excluded from the analysis, and only the excitatory neuron types in each of the six areas were considered.

**Fig 10:**
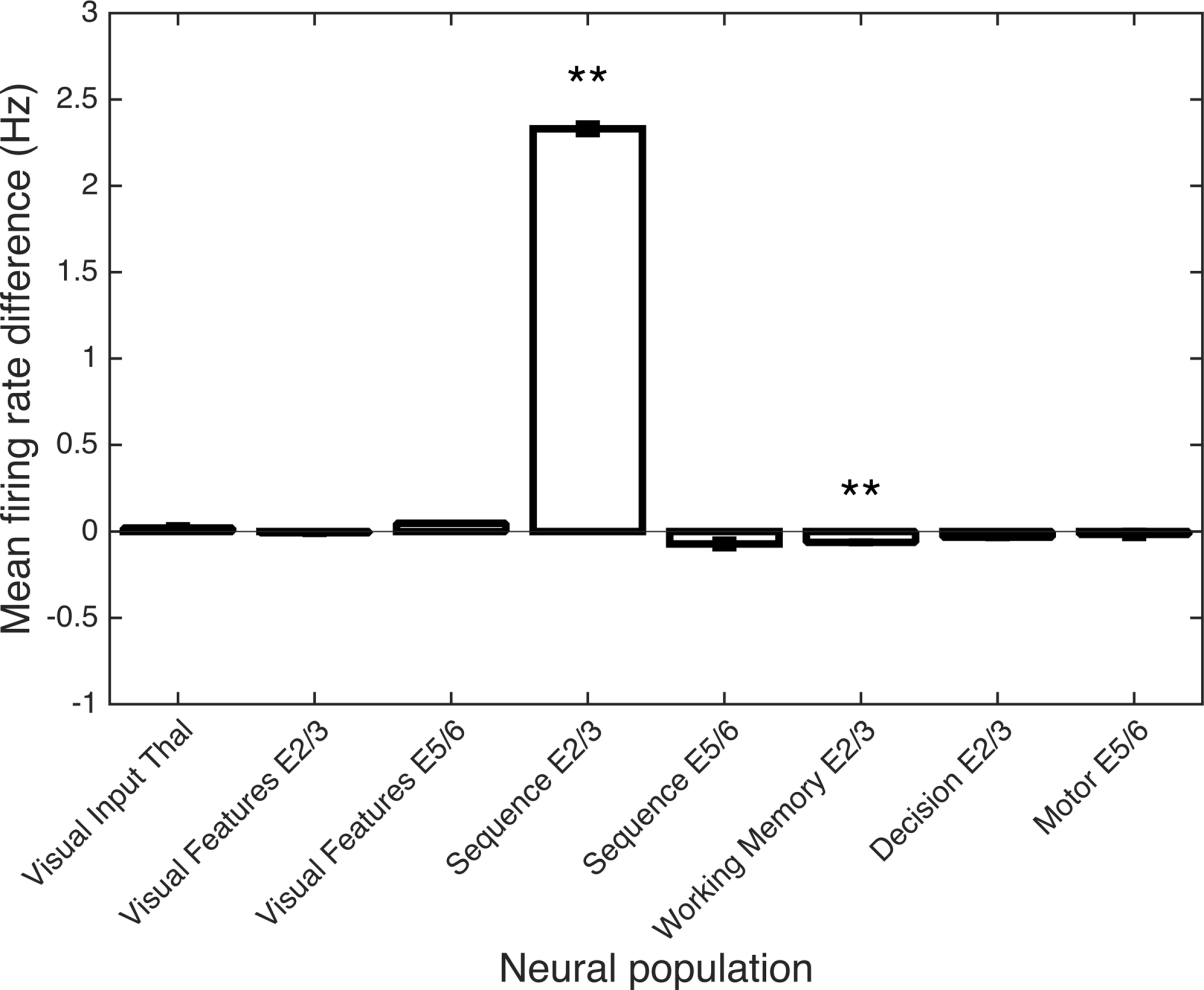
The Sequence area shows increased activity during mental rotation. Mean firing rates were calculated per cell type over the 8 trials for each subject where Stimulus A and B were identical in both object and orientation. The mean firing rates during the mental rotation task were subtracted from the corresponding rates prior to training with rotating objects. The primary difference was in the Sequence area E2/3 neurons, which were cycling through the activity patterns corresponding to the object orientations only during the mental rotation task. Statistically significant differences (P<0.01, Wilcoxon Rank Sum, N = 5) are indicated by double asterisks.

Fig 10 shows the differences when the mean firing rates in the passive viewing condition were subtracted from those in the mental rotation task. The primary difference is an increased mean firing rate in the E2/3 neurons in the Sequence area (P<0.01, Wilcoxon Rank Sum, N = 5). This increased firing reflects the sequence of activity patterns maintained in the response phase of the mental rotation task. A small but significant difference was also present in the Working Memory area (P<0.01); otherwise the mean firing rates are very similar in the two conditions.

Another approach to assessing the overlap in neural activity in the two conditions is to measure the fraction of cells active in one or both conditions. The results are given in Table 3. The results indicate that the vast majority of cells are active both in passive viewing and in the mental rotation task.

**Table 3:** Comparison of the percentages of neurons active during mental rotation task and during stimulus presentation prior to experience with rotating objects.

Area, Cell Type	% Neurons Active	% Active in Both Conditions	% Active in Viewing Only	% Active in Mental Rotation Only	Activity Correlation Coefficient
Visual Input, Thal	54.0	91.9	2.6	5.5	0.888
Visual Features, E2/3	31.8	96.4	0.5	3.1	0.803
Visual Features, E5/6	28.6	92.8	0.8	6.4	0.760
Sequence, E2/3	39.4	87.0	8.3	4.7	0.625
Sequence, E5/6	34.3	82.7	13.7	3.6	0.805
Working Memory, E2/3	24.3	77.8	9.5	12.7	0.779
Decision, E2/3	21.7	80.4	6.8	12.8	0.807
Motor, E5/6	10.1	93.9	2.3	3.8	0.999

Also, we would expect a significant correlation between a neuron’s firing rate in the two conditions—if a neuron fires vigorously during passive viewing, it should also fire vigorously during the mental rotation task. The results given in Table 3 indicate that the population firing rates in the two conditions are highly correlated within each area, providing additional evidence that the model uses the same machinery during passive viewing and during the performance of a mental imagery task.

## Discussion

In this paper we extended the idea that mental imagery may arise from associations [9] and proposed that sequence learning may form the basis of the ability to perform mental rotation. To prove the feasibility of this approach, our goal was to test whether a neural model consistent with key biological principles, that is capable of sequence learning could perform a task analogous to mental rotation. By observing a rotating object, Darwin XIII learns the sequence of neural activity patterns generated by the visual system responding to the object’s rotation. After learning, it can internally regenerate a similar sequence of neural activations upon briefly viewing the static object. When performing a DMS version of the mental rotation task, the model compared the neural activity from the first stimulus with a regenerated activity sequence initiated by the second stimulus, thus “filling-in” intermediate angles as is hypothesized to occur during mental rotation in humans. Only after learning about rotation through experience could Darwin XIII spontaneously report that one stimulus of a pair was a rotated version of the other, and it did so with a reaction time proportional to the angular difference between the stimuli. The results of our simulations demonstrate that, in spite of the simple nature of this model, it matches key behavioral data and functions in a manner consistent with internal cognitive processes [3–6,18].

Filling-in intermediate angles by regenerating a learned sequence of neural activity representing object rotation is consistent with neurophysiological data. While the mental rotation task has yet to be replicated in non-humans [19], single-unit recordings during a related task in primates show that the monkey’s intended direction of movement, as reflected in the motor cortex, changes continuously as the monkey plans a movement 90 degrees away from the direction of the movement cue [20]. Studies of human reaction times [4], priming [5], and fMRI [6] when the subject is performing a task similar to the DMS task described above, all suggest that the brain mentally rotates the object through intermediate angles. We have proposed that this filling-in of intermediate angles occurs via the learning and regenerating sequences of neural activity representing the visual input of a rotating object.

Neural activity occurring during mental imagery in humans is very similar to that occurring when the subject actually sees the imagined stimulus (for a review, see [9]). Similarly, in Darwin XIII during the Response period, in the absence of any stimulus, both the patterns of activity (Fig 7) and the populations of active neurons (Table 3) correspond closely to those seen when the actual stimuli are present.

This work presented here has several limitations. We do not claim that Darwin XIII is actually performing mental rotation as a human does, but rather intend the model as validation of the concept that sequence learning and regeneration could support mental rotation. We limited both the number of objects and the number of orientations used as stimuli in order to avoid having to make fine discriminations in the simulated visual system, as well as to keep the training sequences short. The possible number of objects/orientations that can be encoded is determined by the specific network architecture and parameter choices for the spiking networks used [13,14], but for all reasonable choices of parameters it will be finite and much too small to make fine orientation distinctions among more than a few objects. We also limited the rotation sequence to the counter-clockwise direction in order to simplify the sequence generation network. While the current system is not general-purpose or capable of rotating novel objects, future work could build on the principles defined here to extend such a system in any of these more general directions. For example, the present system learns neural activity patterns that represent the visual inputs as a gestalt or gist of high-level object features; activity does not encode low-level topological features of the object, such as edges, corners, or surfaces. An extension of the model that learned such low-level, generic features could endow the system with the ability to rotate novel objects.

Sequence learning is critical to other tasks besides mental rotation, and it is possible that there may be a common mechanism that subserves sequence learning in many different tasks. In support of this idea, neuroimaging studies have shown significant overlap between brain regions involved in mental rotation [21] and visuomotor sequence learning [22], particularly regions around the intraparietal sulcus (IPS). The posterior parietal cortex has been implicated in mental rotation [21] and in artificial grammar learning [23], a task that is strongly sequence-dependent. There is overlapping activation of parietal cortex in both mental rotation and auditory imagery, which by its nature is sequential [24]. Learning visual Markov sequences also strongly activates the IPS [25]. These results suggest that parietal cortex is involved in sequence learning. Our model suggests that mental rotation arises by the mechanism of sequence learning, a view that is consistent with suggestions that parietal cortex is part of a multi-modal implicit learning system that associates sequences of non-categorized stimuli [11].

Other investigators have been interested in the use of robotically embodied neural models to investigate mental imagery, and a recent special issue collects many examples from this field [26]. Additional neurorobotic models have addressed motor imagery [27], the use of imagery as an aid to learning [28], and multi-modal associative learning to create imagery [29]. Recent work has addressed mental rotation in a mean firing rate neural model that controlled a virtual humanoid robot [30]. In contrast to that work, our model proposes that sequence learning may underlie mental rotation, uses a spiking neural model with conductance-based synapses, and controls a physical humanoid robot. Because sequence learning is a general phenomenon that could be applied to any sequence of perceptions, it seems likely that our model could be extended to many of the same tasks that are addressed in the citations above.

Our results suggest several testable predictions. First, our implicit assumption is that adult human subjects should be able to perform our DMS version of the mental rotation task, and we predict that one would find the classic linear dependence between response time and orientation difference.

Second, we predict that without experience viewing objects rotating, subjects will be unable to perform the mental rotation task. Animal experiments could potentially falsify this conjecture, by dark rearing animals to avoid visual rotation exposure, followed by training on a DMS task, and then looking for spontaneous generalization after experience with rotating objects. This prediction could also be tested in congenitally blind human subjects whose sight has been restored [31].

Third, while the handling of an object and manually rotating it would perhaps be helpful, behavioral data in human subjects shows that it is not required [32]; see [33] for an alternative perspective). Therefore we predict that humans who never had the ability to physically manipulate objects, but who did have appropriate visual experiences, should still be able to perform the task with the same dependence between time and orientation difference.

Fourth, since in our model mental rotation is a special case of learning and regenerating a sequence, we predict that reaction times proportional to a difference of sequence position could arise for learned visual sequences that have nothing to do with physical rotation. If a subject has sufficient experience with arbitrary visual sequences to be able to internally visualize them, then the time taken to report whether two images belong to the same sequence should be proportional to the sequence position difference on match trials. Activation of the parietal cortex may also be revealed by fMRI during such an experiment.

Finally, while imagery is a conscious experience, our model suggests that it may be based on sequence learning that takes place without awareness. It has been proposed that a general purpose implicit learning system for sequences exists that includes parietal cortex [11], an area known to be involved in mental rotation [21]. The replay of implicitly learned sequences could also be expressed without awareness, similar to other mental processes [34]. We therefore predict that use of a distractor task would reveal that mental rotation could occur without awareness but with the same linear reaction time signature.

Albright [9] recently suggested that learned associations may link perception and imagery. Perceived stimuli must interact with stored memories to allow the brain to interpret ambiguous sensory signals. Neurophysiological evidence for the hypothesis came from his laboratory’s experiments in which associations formed between the direction of moving dots and a static arrow pointed up or down imparted motion-sensitive cells in area MT with selectivity for static arrows. Surprisingly, synesthesia can be acquired through training [35], which also supports this idea. Our model shows that such Hebbian associative learning can explain how the ability to generate mental rotation may arise in the networks of the brain. Furthermore, the model demonstrates that, consistent with Albright’s hypothesis, these learned associations allow for the interpretation of sensory signals. In fact, Darwin XIII was not able to equate the different orientations of the same stimulus until sequential associations were learned through experience. Stored memories of rotating objects allowed Darwin XIII to spontaneously interpret the associated stimuli as equivalent, by “going beyond the information given” by the stimulus [10]. The simulations support the notion that associations learned from sensory experiences may give rise to mental imagery.

Mental imagery may be a necessary byproduct of a neural system that faithfully models the external world in support of internal simulations useful for prediction, interpreting ambiguity, and planning [2,36]. Our system learns a model of the visual input stream through observation, and this mental model faithfully reflects one aspect of the external world: the relationship between rotation angle and time. Creating systems capable of increasingly complex, learned internal simulations of the external world may lead to a better understanding of not only how mental imagery arises, but also the bases of other higher brain functions [37].
